# Effects of Palm Oil Tocotrienol-Rich Fraction (TRF) and Carotenes in Ovalbumin (OVA)-Challenged Asthmatic Brown Norway Rats

**DOI:** 10.3390/ijms20071764

**Published:** 2019-04-10

**Authors:** Zaida Zainal, Afiqah Abdul Rahim, Huzwah Khaza’ai, Sui Kiat Chang

**Affiliations:** 1 Malaysian Palm Oil Board, Bandar Baru Bangi, Selangor 43000, Malaysia; afiqah@mpob.gov.my; 2 Department of Biomedical Sciences, Faculty of Medicine and Health Sciences, Universiti Putra Malaysia, Serdang 43400, Selangor, Malaysia; huzwah@upm.edu.my; 3 Department of Nutrition and Dietetics, School of Health Sciences, International Medical University, Bukit Jalil 57000, Kuala Lumpur, Malaysia; suikiatchang@gmail.com

**Keywords:** OVA-induced asthma model, palm oil, palm tocotrienol-rich fraction, carotene, antioxidant, inflammation

## Abstract

Synthetic therapeutic drugs for asthma, a chronic airway inflammation characterised by strong eosinophil, mast cell, and lymphocyte infiltration, mucus hyper-production, and airway hyper-responsiveness, exhibit numerous side effects. Alternatively, the high antioxidant potential of palm oil phytonutrients, including vitamin E (tocotrienol-rich fractions; TRF) and carotene, may be beneficial for alleviating asthma. Here, we determined the therapeutic efficacy of TRF, carotene, and dexamethasone in ovalbumin-challenged allergic asthma in Brown Norway rats. Asthmatic symptoms fully developed within 8 days after the second sensitization, and were preserved throughout the time course via intranasal ovalbumin re-challenge. Asthmatic rats were then orally administered 30 mg/kg body weight TRF or carotene. TRF-treated animals exhibited reduced inflammatory cells in bronchial alveolar lavage fluid. TRF- and carotene-treated rats exhibited notable white blood cell reduction comparable to that from dexamethasone. TRF- and carotene-treatment also downregulated pro-inflammatory markers (IL-β, IL-6, TNF-α), coincident with anti-inflammatory marker IL-4 and IL-13 upregulation. Treatment significantly reduced asthmatic rat plasma CRP and IgE, signifying improved systemic inflammation. Asthmatic lung histology displayed severe edema and inflammatory cell infiltration in the bronchial wall, whereas treated animals retained healthy, normal-appearing lungs. The phytonutrients tocotrienol and carotene thus exhibit potential benefits for consumption as nutritional adjuncts in asthmatic disease.

## 1. Introduction

Asthma constitutes a chronic inflammatory airway disease that is characterized by bronchial hyper-responsiveness owing to different stimuli, bronchoconstriction, airflow restriction, and inflammation of the bronchi causing coughing, wheezing, chest tightness, and dyspnea [1,2]. Airflow obstruction in asthma is preceded by bronchoconstriction, followed by mucosal edema, increased secretion of mucus, and infiltration of inflammatory cells into smooth muscle tissues with abundance of eosinophils in the bronchial mucosa, leading to excessive mucus secretion [3]. Asthma represents a common medical condition seen at all levels of health care in Malaysia. Moreover, according to the latest World Health Organization (WHO) data, published in 2017, asthma deaths in Malaysia reached 1258, accounting for 0.91% of total deaths, with an age adjusted death rate of 5.10 per 100,000 population [4].

Factors involved in the pathogenesis of asthma include quantities of inflammatory cells, mediators, nerves, and vascular leakage. Allergic asthma is caused by allergens such as dust, pollen, and animal dander that trigger inflammation and initiate airway remodeling, which is characterized by deposition of reconditioned collagens, along with matrix protein damage owing to continuous inflammation and damage of the airway epithelium [2,5]. Specifically, Th2 lymphocytes promote the release of interleukin (IL)-5 by eosinophils and IL-4 via immunoglobulin E (IgE) in mast cells [6]. Thus, the amount of serum IgE increases, inducing mast cells to synthesize more cytokines and leukotrienes [7] along with chemical mediators such as histamine [8]. Histamine promotes bronchoconstriction, increasing vascular permeability and finally producing more mucus, whereas prostaglandin D2 triggers bronchoconstriction and leukotrienes concomitantly enhance vascular permeability, followed by mucus secretion and bronchoconstriction [9]. This late reaction includes the enrolment of mast cell and other effector cells, especially TH2 lymphocytes, eosinophils, neutrophils, and basophils, which are characteristic of asthma pathogenesis. Accordingly, corticosteroids are commonly prescribed to alleviate asthmatic conditions [10], however, these elicit various undesirable side effects. Long term consumption of asthma controlled medications, such as anti-IgE or immunomodulators, by asthmatic patients was reported to cause development of anaphylaxis, platelet dysfunction, GI problems, acute renal failure, and edema [10].

As an alternative, several lines of evidence have indicated that antioxidants may provide a rational therapeutic approach for asthma [11,12,13]. Vitamin E and vitamin A carotenoids constitute good antioxidants that might be considered for their therapeutic effects in asthma. Animal experiments carried out by Jiang et al. [14] demonstrated that supplementation of mice with alpha-tocopherol diminished allergen-induced airway inflammation and airway responsiveness. Moreover, a clinical study by Ghaffri et al. [15] revealed that vitamin E supplementation in children with moderate asthma could enhance clinical indications and respiratory test results in this population.

Malaysia constitutes one of the world’s largest producers and exporters of palm oil, which is commonly used by Malaysians in cooking. Palm oil contains high levels of tocotrienols, with crude palm oil containing up to 800 mg/kg of α- and γ-tocotrienols. Moreover, palm oil carotenes comprise 13 forms, including α-carotene, β-carotene, γ-carotene, lycopene, and other carotenoids [16], representing a novel combination of carotenes that is usually obtained from fruits and vegetables. The ratio of α-carotene and β-carotene in palm oil carotene is 35% and 60% [17], respectively, which exactly matches the carotenoid content found in carrot. In comparison, vitamin E can be divided into two isoforms, tocopherols and tocotrienols, with the distribution in palm oil being approximately 30% tocopherol and 70% tocotrienols [18]. These consist of eight different forms: the α,β, γ, and δ-tocopherols, along with the α, β, γ, and δ-tocotrienols. All eight forms function as antioxidants, capable of scavenging and quenching free radicals by donating an electron to neutralize the reactive free radicals [19], although tocotrienols have been shown to be more efficient than tocopherols at scavenging peroxyl radicals [20]. Consistent with this observation, numerous studies have revealed the potential of tocotrienols in reducing the risk of heart disease, increasing brain health and functionality, and promoting anticancer activity.

In turn, the aim of this study was to determine the lung-protective effects of palm oil tocotrienol-rich fraction (TRF) and carotene supplementation on asthmatic inflammation, using the ovalbumin-challenged (OVA) asthmatic rat model. In addition, the effects of palm oil TRF and carotenes on the mediators of pro- and anti-inflammatory cytokines were examined in experimental rats. Most studies have demonstrated that pre-treatment approach in asthma using vitamin E. However, none of the work has reported on the TRF and carotene in the treatment after the onset of asthma. Notably, the benefits of TRF and carotene observed on imaging may serve to reduce the dependency on anti-asthma drugs, thereby reducing the associated side effects, along with improving the symptoms of asthma.

## 2. Results

### 2.1. Clinical Presentation of Asthma upon OVA-Challenge in Experimental Rats

**Figure 1:**
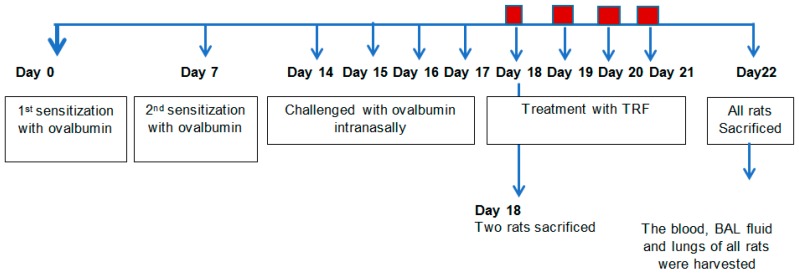
Study design. The time course for treatment protocols is summarized.

The experimental design for OVA challenge and treatment with TRF or carotene is shown in Figure 1. In asthma model rats, asthmatic symptoms eventually developed around day 8 after the second sensitization through day 17, which represented the end of four consecutive days of intranasal OVA-challenge. The rats were observed daily throughout the study to identify any uncommon behavior. Upon OVA-challenge, rats were inactive, presenting with rhinorrhea and rapid breathing. They showed noticeable respiratory discomfort, represented by defensive action and labored breathing movements. The OVA-challenged rats exhibited wheezing, dyspnea, and coughing, especially at night and early in the morning, along with breathlessness and increased mucus production. Furthermore, respiratory OVA-challenged rats demonstrated increased expiration relative to inspiration. Consequent to rapid onset attack, they breathed through their mouth. Symptoms were worse at night and limitation of activity of the OVA-challenged rats was observed compared to that of the control, TRF, and carotene treated rats. Animals treated with TRF or carotene did not demonstrate any respiratory distress.

### 2.2. Effects of TRF and Palm Carotene on the Body Weight of OVA-Challenged Asthma Model Rats

**Figure 2:**
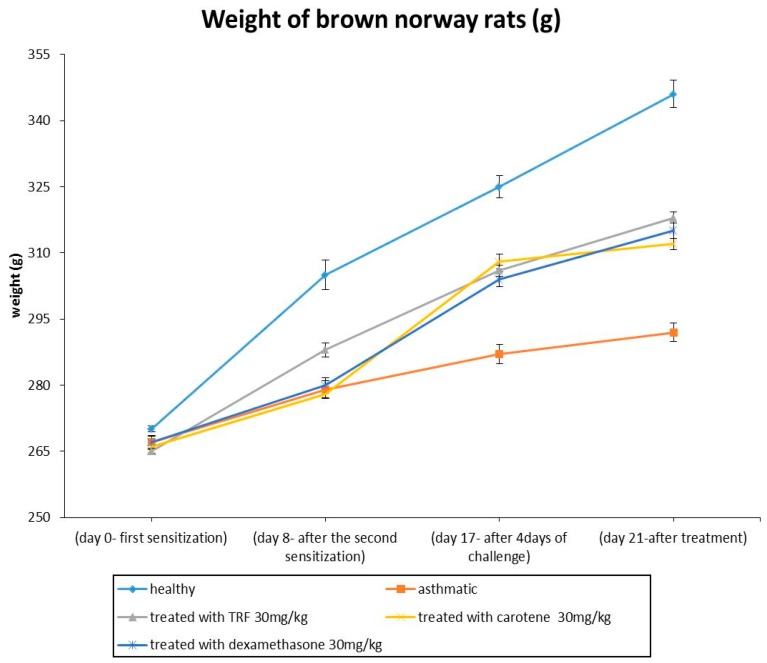
Body weights between healthy, ovalbumin (OVA)-challenged, and tocotrienol-rich fractions (TRF) (30 mg/kg body weight), carotene (30 mg/kg body weight) + OVA, and dexamethasone (30 mg/kg body weight) + OVA treated rats. Values are expressed as the mean ± SEM (𝑛 = 10). ^a^ (*P* < 0.05) versus the control group. ^b^ 𝑃 < 0.05, significantly different from the OVA-challenged group. Note that OVA-sensitization was effected via the intraperitoneal (IP) method, whereas OVA challenge was performed intranasally.

The disease progression was monitored by examining the body weight changes throughout the study period. The body weight of the healthy rats increased significantly with time compared with that of other groups (Figure 2). The TRF-, carotene-, and dexamethasone-treated rat groups exhibited body weight increase after the first OVA-challenge until sacrifice compared to that of the asthmatic groups. Minimal body weight increase was observed in asthmatic rats as compared to that of the healthy group.

### 2.3. Effect of TRF and Palm Oil Carotene on Eosinophil, Lymphocyte, Neutrophil, and Monocyte Cell Morphology

**Figure 3:**
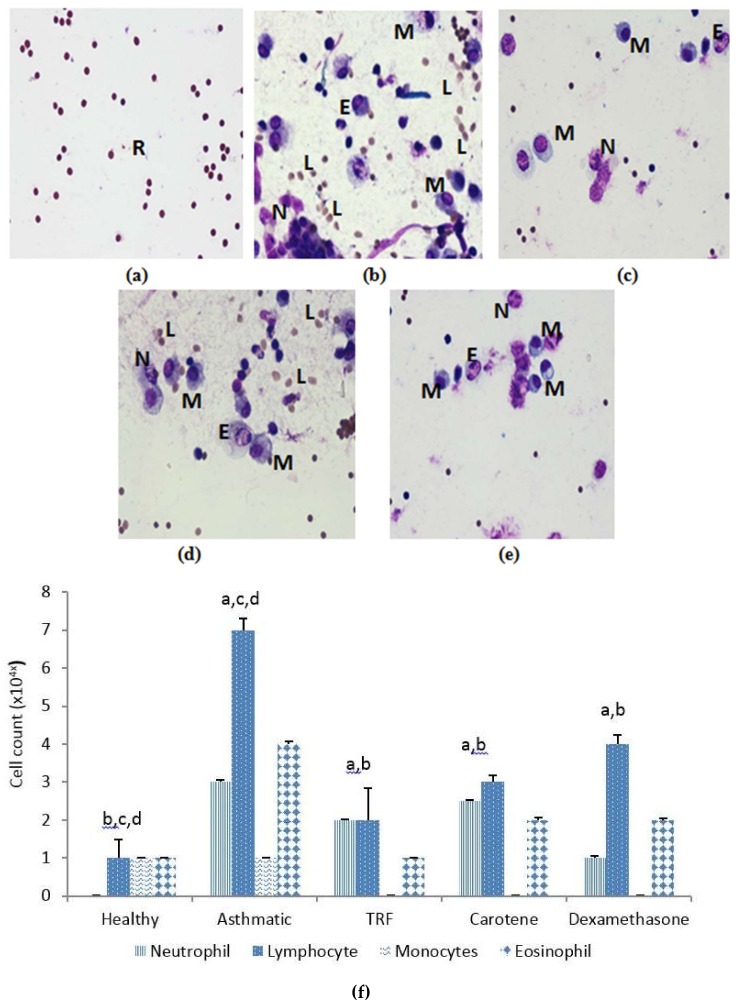
Staining of inflammatory cells from broncho-alveolar lavage (BAL) fluid samples. (**a**) Healthy sample; (**b**) asthmatic sample (+ OVA); and samples treated with TRF (30 mg/kg) + OVA (**c**); carotene (30 mg/kg) + OVA (**d**); and dexamethasone + OVA (**e**). N, neutrophil; E, eosinophil; L, lymphocyte; M, monocyte; R, red blood cell. High magnification 100×. (**f**). The inflammatory cell count in the BAL fluid samples. The values are expressed as means ± SD (𝑛 = 10). ^a^ *P* < 0.05 versus the control group. ^b^ *P* < 0.05, significantly different from the OVA-challenged group, ^c^ *P* < 0.05 versus OVA + TRF, ^d^ *P* < 0.05 versus OVA + dexamethasone.

Whole blood analysis and cytology of broncho-alveolar lavage (BAL fluid) was conducted to elucidate the responsiveness of inflammatory cells upon OVA sensitization and treatment with palm oil TRF and carotene. Eosinophils, neutrophils, and especially lymphocytes exhibited substantive decrease after treatment with palm oil TRF and carotenes, compared to those of OVA-challenged rats. The healthy rats (group A) were free from inflammatory cells, which confirmed that the rats were non-asthmatic compared to the OVA-challenged group (Figure 3a–e). The reduced number of inflammatory cells following treatment with TRF and carotenes was comparable to that obtained with dexamethasone. However, palm oil carotene showed negligible effect in reducing inflammatory cells in BAL fluid (Figure 3f).

### 2.4. Effect of Palm Oil TRF and Carotenes on White Blood Cell Count

**Figure 4:**
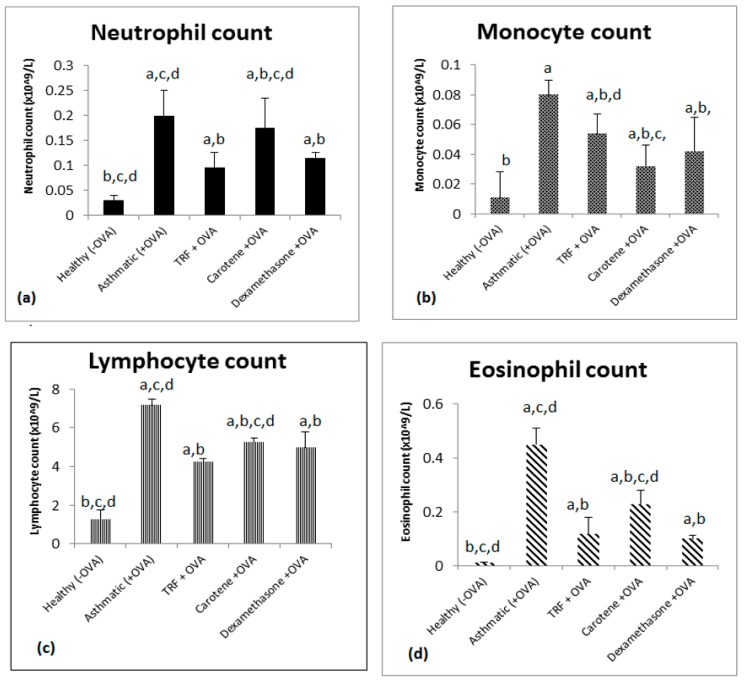
Effect of palm oil TRF, carotene, and dexamethasone on the total and differential cell count in whole blood of OVA-challenged asthmatic rats. The number of (**a**) total neutrophils, (**b**) lymphocytes, (**c**) monocytes, and (**d**) eosinophils from whole blood. The values are expressed as the means ± SD (𝑛 = 10). ^a^ *P* < 0.05 versus the control group. ^b^ *P* < 0.05, significantly different from the OVA-challenged group, ^c^ *P* < 0.05 versus OVA + TRF, ^d^ *P* < 0.05 versus OVA + dexamethasone.

Figure 4 shows that the cell counts of eosinophils, lymphocytes, monocytes, and neutrophils in whole blood were increased in the OVA-challenged asthmatic group compared with those of the healthy or control groups. Treatment with palm oil TRF and carotene (30 mg/kg body weight) significantly reduced the number of white blood cells compared to that in the asthmatic rats. Notably, palm oil TRF more effectively reduced the cell count of neutrophils, lymphocytes, and eosinophils (Figure 4a,b,d), whereas carotenes were more effective in lowering the cell count of monocytes (Figure 4c). Palm oil TRF was also more effective at lowering the cell count of neutrophils and lymphocytes than dexamethasone (Figure 4a,b), even though the differences were not significant (*P* > 0.05). In comparison, carotenes significantly reduced the monocyte cell count compared to that of dexamethasone (Figure 4c; *P* < 0.05).

### 2.5. Effect of Palm Oil TRF and Carotenes on Proinflammatory Cytokines

**Figure 5:**
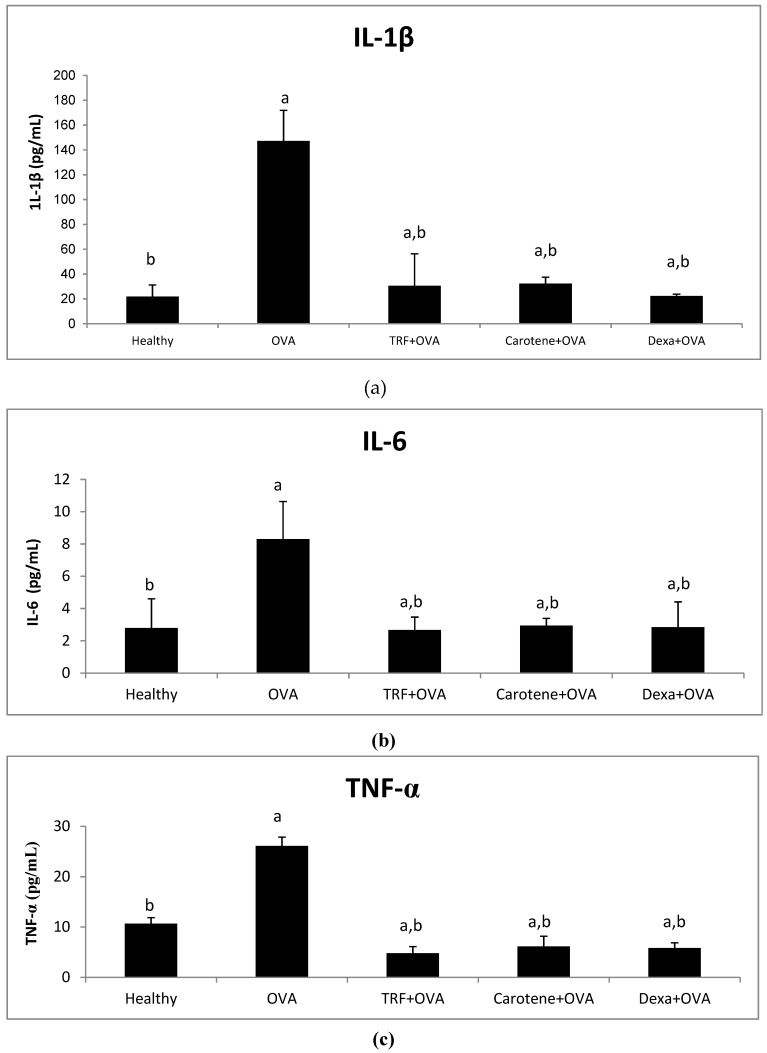
Effects of palm oil TRF, carotenes, and dexamethasone on pro-inflammatory cytokines, (**a**) IL-1β, (**b**) IL-6, and (**c**) TNF-α, in the plasma of Brown Norway rats challenged with OVA. Data are presented as the means ± SD, *n* = 10 rats per group. ^a^ *P* < 0.05 versus the control group. ^b^ *P* < 0.05 versus the OVA-challenged group.

Figure 5 shows that the concentrations of IL-1β, IL-6, and TNF-α were markedly elevated in the OVA-challenged rats compared with those in controls (*P* < 0.05). Palm oil TRF and carotene treatments reduced serum levels of IL -1β by 77% and 78%, IL-6 by 5.67% and 5.36%, and TNF-α by 81.6% and 76.3% compared to those of the control groups, respectively (Figure 5). Treatments with dexamethasone significantly decreased all the pro-inflammatory cytokines in the plasma. No significant difference (*P* > 0.05) was observed between the effects of palm oil TRF and carotenes with regard to reducing the pro-inflammatory cytokines (Figure 5), indicating that the effects of palm oil TRF and carotenes were comparable to those of the drug dexamethasone.

### 2.6. Effect of Palm Oil TRF and Carotenes on Anti-inflammatory Cytokines

**Figure 6:**
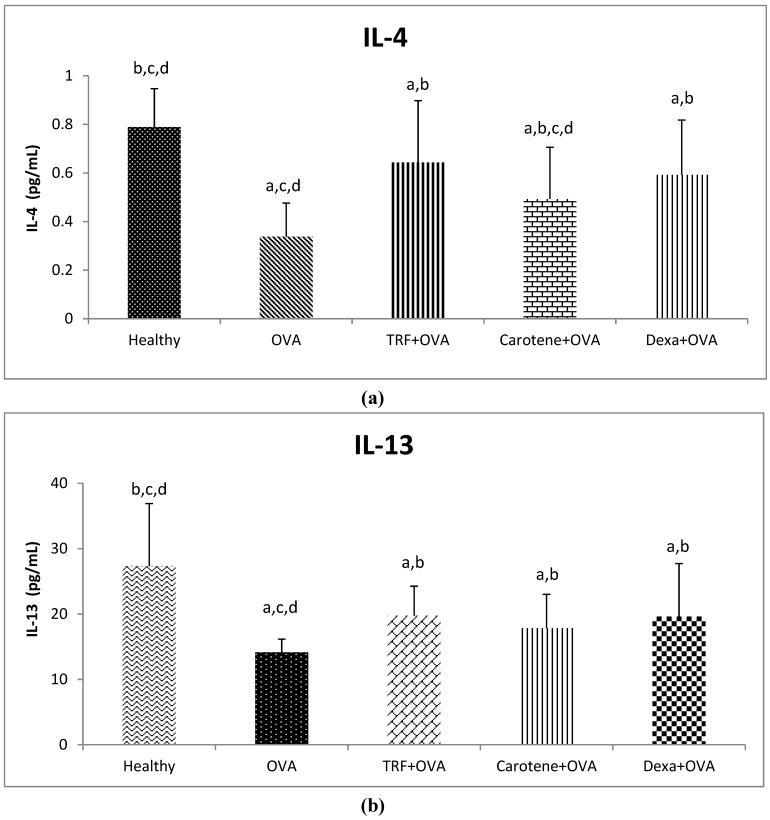
Plasma concentrations of (**a**) IL-4 and (**b**) IL-13 after treatment with palm oil TRF, carotenes, and dexamethasone (Dexa). Data are presented as the means ± SD, *n* = 10 rats per group. ^a^ *P* < 0.05 versus the control group, ^b^ *P* < 0.05 versus the OVA-challenged group, ^c^ *P* < 0.05 versus OVA + TRF, ^d^ *P* < 0.05 versus OVA + Dexa.

Figure 6 shows that the levels of IL-4 and IL-13 in the plasma were significantly downregulated following challenge with OVA. However, IL-4 and IL-13 levels were significantly upregulated in the rat groups treated with palm oil, TRF, carotene, and dexamethasone (Figure 6a,b). Palm oil TRF and carotene treatments increased serum levels of IL-4 by 85% and 30% and IL-13 by 39% and 26% compared to those of the control groups, respectively, whereas dexamethasone increased the IL-4 and IL-13 levels by 71% and 38.6% (Figure 6a,b). Notably, the effects of palm oil TRF and dexamethasone on plasma IL-4 and IL-13 did not significantly differ (*P* > 0.05), indicating that TRF is effective in increasing anti-inflammatory cytokines.

### 2.7. Effect of Palm Oil TRF and Carotenes on Plasma C-Reactive Protein (CRP) and IgE Levels

**Figure 7:**
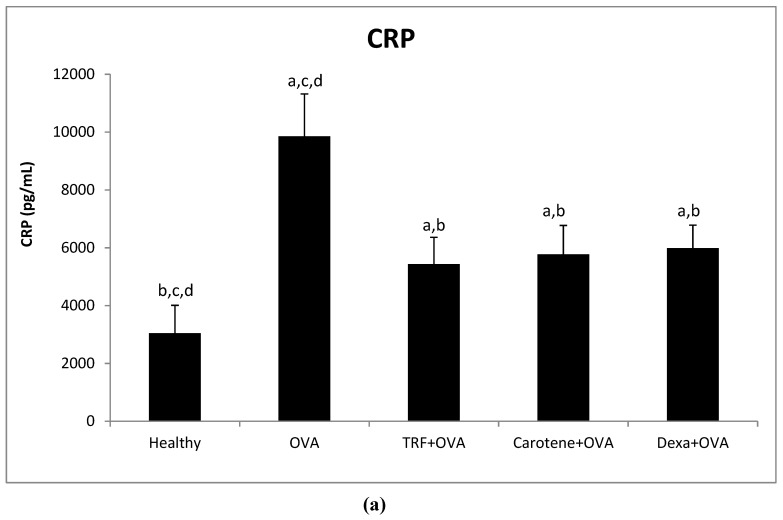

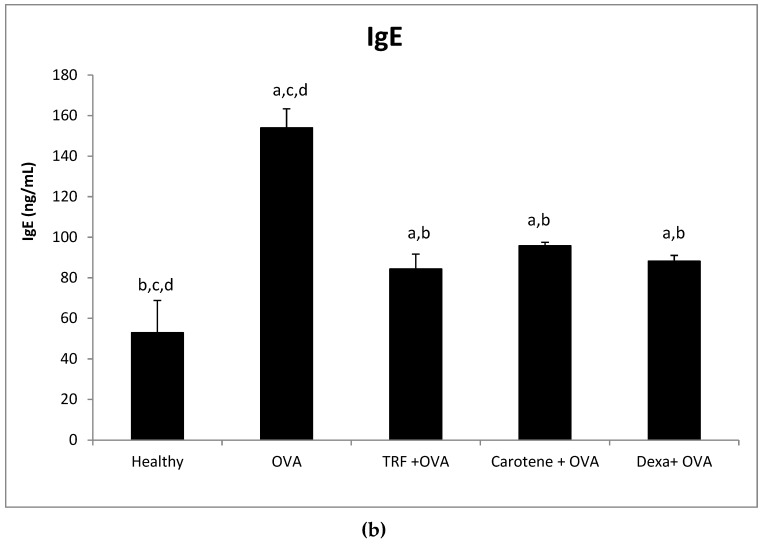
Plasma concentrations of (**a**) CRP and (**b**) IgE after treatment with oil palm TRF, carotenes, and dexamethasone (Dexa). Data are presented as the means ± SD, n = 10 rats per group. ^a^ (*P* < 0.05) versus the control group. ^b^ *P* < 0.05 versus the OVA challenged group ^c^ *P* < 0.05 versus OVA + TRF, d *P* < 0.05 versus OVA + Dexa.

As shown in Figure 7, plasma CRP and IgE levels were significantly increased in OVA-challenged asthmatic rats. Plasma CRP level was decreased by 44%, 41%, and 39% in rats treated with palm oil TRF, carotene, and dexamethasone, respectively, compared with that in the OVA-challenged group. However, there was no significant difference between these two treatments and dexamethasone, although both treatments showed greater relative reduction in plasma CRP levels (Figure 7a). Plasma CRP levels in the OVA-challenged group were significantly (68%) higher than those in healthy rats. Moreover, treatment with palm oil TRF, carotenes, and dexamethasone reduced plasma IgE levels significantly compared to those of OVA-challenged asthmatic rats (Figure 7b).

### 2.8. Effects of Palm Oil TRF and Carotenes on the Leukotrines LTB-4 and LTB-5

**Figure 8:**
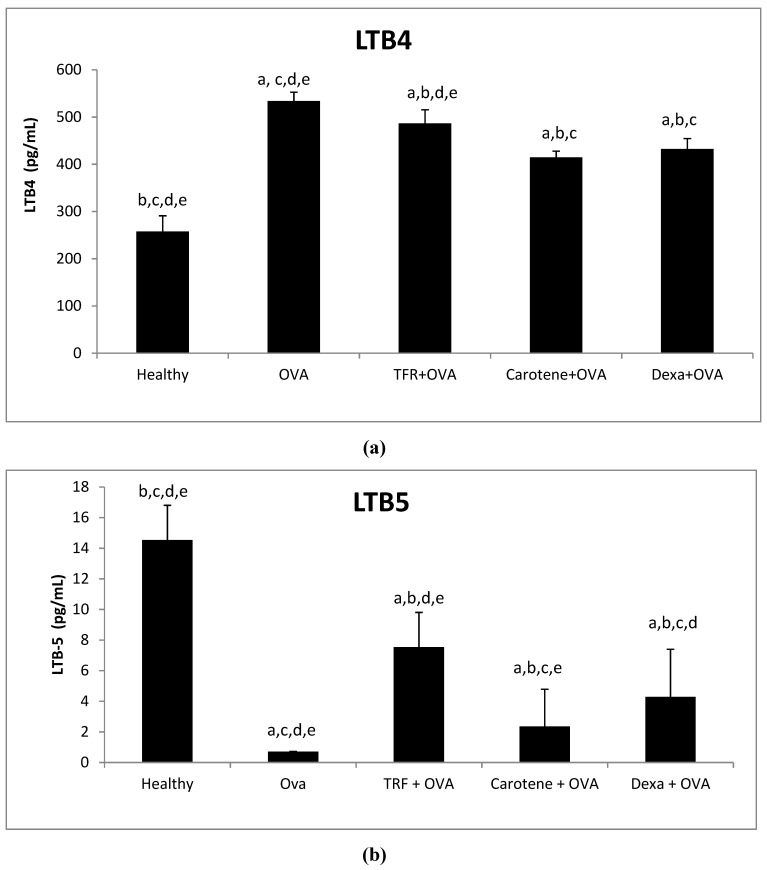
Plasma concentrations of the leukotrienes (**a**) LTB-4 and (**b**) LTB-5 after treatment with palm oil TRF, carotenes, and dexamethasone (Dexa). Data are presented as the means ± SD, *n* = 10 rats per group. ^a^ *P* < 0.05 versus the control group, ^b^ *P* < 0.05 versus the OVA challenged group, ^c^ *P* < 0.05 versus OVA + TRF, ^d^ *P* < 0.05 versus OVA+ Carotene, ^e^ I < 0.05) versus OVA + Dexa.

The levels of LTB-4 were significantly downregulated by 8.8%, 22.3%, and 19.0% after treatment with palm oil TRF, carotenes, and dexamethasone compared with those in OVA-challenged asthmatic rats, (Figure 8a). Conversely, serum levels of LTB-5 were upregulated in the healthy and treated rats compared to those in OVA-challenged asthmatic rats (Figure 8b). Treatment with palm oil TRF and carotenes significantly upregulated LTB-5 level by 92% and 75%, respectively, compared to that of OVA-challenged rats. In comparison, the LTB-5 level of rats was upregulated by 83% after treatment with dexamethasone as compared to that in OVA-challenged asthmatic rats (Figure 8b).

### 2.9. Effect of Palm Oil TRF and Carotenes on Lung Histology

**Figure 9:**
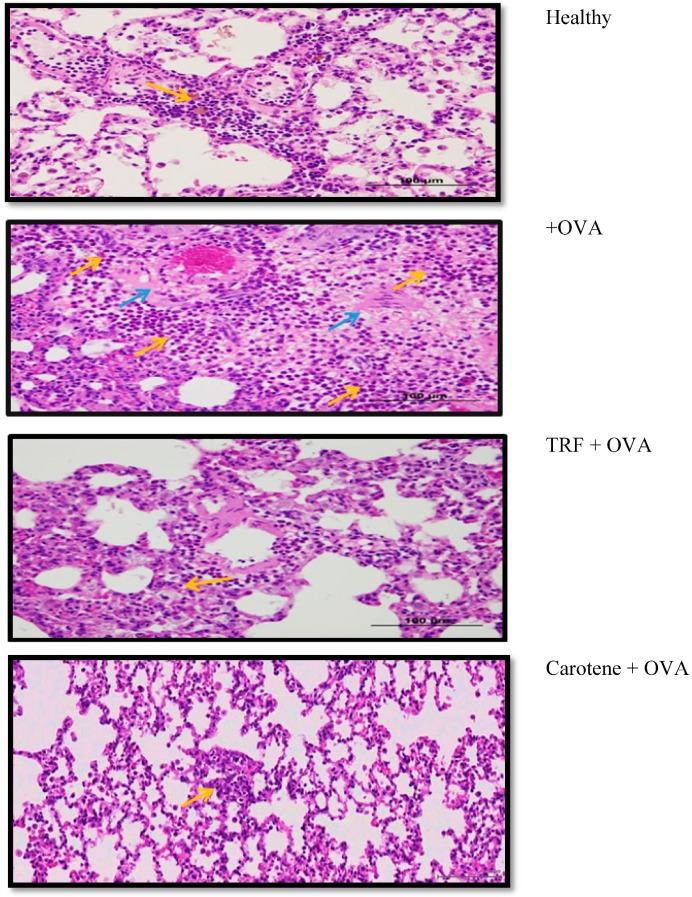

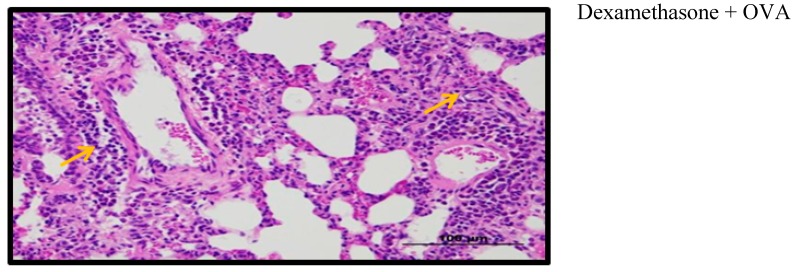
Histology of the polymorphonuclear and mononuclear cell infiltrate the various treated rat groups. Histology in the healthy rat lung was within normal limits (less inflammatory cells), whereas the TRF-treated rat showed a notable decrease in inflammatory cells compared to those of the other groups. Yellow arrows indicate polymorphonuclear and mononuclear cell infiltrate; blue arrows indicate giant cell infiltrate.

A total of five lungs (healthy, asthmatic, TRF, carotene, and dexamethasone) from rats administered the different treatments were evaluated histopathologically. In the asthma-induced rats, multifocal areas of intense infiltration of polymorphonuclear cells, macrophages, giant cells, lymphocytes, and plasma cells were observed, which filled the alveolar spaces and extended to the peribronchial, perivascular, and interstitial spaces. In addition, perivascular edema and increased numbers of alveolar macrophages were detected in the lungs. Regions of alveolar hemorrhage and hemosiderin-laden macrophage infiltrate were also present in OVA-challenged rats. In contrast, rats treated with TRF showed less severe destruction, with moderate bronchus-associated lymphoid tissue (BALT) hyperplasia and minimal diffuse perivascular edema with very few macrophages (Figure 9). Only slight decrease of polymorphonuclear, macrophage, and lymphocyte cells was observed in carotene-treated rats, indicating that TRF manifested relatively good improvement with regard to degree of inflammation compared to that of carotene.

## 3. Discussion

The airway inflammation in asthma occurs together with oxidative stress [21], which triggers oxidative damage of proteins, DNA, and lipids, thereby damaging the epithelial cells of the respiratory tract. Accordingly, agents with strong antioxidant ability, such as, vitamins A and E, have been suggested as having potential utility for the prevention of asthma [22].

In the present study incorporating an animal model of asthma, female Brown Norway rats were injected with OVA from chicken egg to stimulate an immunological hypersensitivity effect of collagen, leading to the development of upper respiratory tract inflammation in the rats to induce a strong pulmonary infection. OVA-challenged rats showed markedly higher breathing rate, along with sneezing, irritability, and lethargy. Treatment with palm oil TRF and carotenes protected against asthma by increasing the airway hypo-responsiveness and breathing rate, along with lowering tidal volume. These results indicated the good ability of antioxidants from palm oil TRF and carotenes to stimulate a bronchodilator effect, which constitutes the main objective in the therapy for asthma. Moreover, these results were almost comparable with those of the commercial drug, dexamethasone. In addition, the body weight of animals treated with TRF, carotene, and dexamethasone increased significantly, indicating the improvement of asthmatic symptoms compared to those of the induced asthmatic animals. These findings are consistent with prior report that dexamethasone consumed as medication for autoimmune diseases and as an anti-inflammatory drug might increase weight gain [23].

Examination of BAL fluid comprises a safe and sensitive procedure to verify diffused pulmonary inflammation at the peripheral airways and alveoli. By conducting cytology staining, the cell count in BAL fluid could distinctly differentiate between healthy, untreated, and treated groups. OVA-challenged asthmatic rats exhibited a multiplication of total cell count compared to that of treated and healthy groups. Fewer inflammatory cells were observed in rats treated with palm oil TRF and carotenes. Notably, lymphocytes, eosinophils, and neutrophils are indispensable for the pathogenesis of asthma [24]. Fahy et al. [25] demonstrated that higher numbers of lymphocytes, monocytes, eosinophils, and neutrophils are detected in patients with asthma. In contrast, our findings demonstrated that the cell counts of all these cells decreased significantly after treatment with palm oil TRF and carotenes. These results are consistent with a study by Wagner et al. [26], who showed that treating OVA-sensitized Brown Norway rats with γ-tocopherol, a potent antioxidant, decreased the accumulation of eosinophils in the septum.

Mediators, including pro-inflammatory cytokines and various chemokines, participate in the pathogenesis of asthma and have become key foci for new therapeutic approaches in curing this disease. Our results verified that TRF and palm carotene significantly suppressed the production of pro-inflammatory markers IL-β, IL-6, and TNF-α in OVA-challenged asthmatic rats. This is consistent with the study by Shen et al. [27], which showed that δ-tocotrienol, isolated from rice bran, significantly inhibited pro-inflammatory cytokine (TNF-α, NF-ƙB, IL-1β, and IL-6) production in lipopolysaccharide-induced macrophages. A previous study also revealed that the existence of Th2 cytokines in an OVA-challenged asthmatic model was critical for the continuous airway inflammation [28]. The current study thus indicated that TRF and palm carotene could function as phytonutrients to counteract the inflammatory response in asthma though their antioxidant effects on Th2-derived pro-inflammatory cytokines.

In accordance with the decrease in pro-inflammatory markers, the present study also identified upregulation of the anti-inflammatory marker LTB-5 in the OVA-challenged asthmatic rat group. Similarly, Jiang and Yin [29] also found that natural forms of vitamin E inhibited LTB-4 in cancer cells.

CRP constitutes an inflammatory marker comprising an important inflammation-sensitive plasma protein generated by the liver [30], with the level of CRP increasing in response to inflammation coincident with IL-6 excretion by macrophages and T cells [31]. The present findings provide evidence that TRF and carotene exhibited strong effects in decreasing the CRP in OVA-challenged rats compared to those in the untreated group. Thus, CRP theoretically may serve as a valuable tool for identifying systemic inflammation in asthma [32].

Elevated levels of IgE were observed in OVA-challenged asthmatic rats, indicating airway hyper-responsiveness that triggered bronchoconstriction, increased vascular permeability, and mucus production. In contrast, downregulation of IgE production was observed in animals treated with palm oil TRF and carotene. Dietary vitamin E intake has been reported to reduce the levels of IgE, and suggested a protective effect of vitamin E against asthmatic disease [33]. Increased production of IgE in allergic asthma constitutes the strongest detectable predisposing factor in the development of bronchial asthma. Noticeable reduction in IgE levels was also detected in the dexamethasone-treated rat group. In comparison, Peh et al. [6] reported that γ-tocotrienol significantly decreased IgE levels in BALB/c mice sensitized by house dust mites. However, this study utilized a high amount of γ-tocotrienol (approximately 250 mg/kg in mice), whereas the present study administered only 30 mg/kg of TRF in the rat asthmatic model.

The polymorphonuclear and mononuclear cell infiltrate in the lung of healthy rats was within normal limits (background lesion); in comparison, the rats treated with TRF showed significant decrease in the number of inflammatory cells compared to those in the other groups. Conversely, in OVA-challenged asthmatic rats, thickening of the bronchial epithelium was observed, along with edema and inflammatory cell infiltrate in bronchial walls. Increase in size of the submucosal mucous glands and hypertrophy of bronchial wall muscle was also detected. Rats in the OVA-challenged group exhibited the most severe inflammation, whereas healthy rats and those treated with TRF and carotenes retained healthy and normal-appearing lungs.

In conclusion, the phytonutrients used in the current study, TRF and palm oil carotene, might serve an important role in the management of bronchial asthma by protecting against the ongoing inflammatory processes underlying asthma through the inhibition of cytokine production. Noticeable increase of anti-inflammatory markers and decrease of pro-inflammatory markers indicated the improvement of lung function in animals treated with TRF and carotene, counteracting the allergen-induced bronchial hyper-responsiveness and blocking the inflammatory cell infiltration (eosinophils, lymphocytes, and neutrophils) into airways. TRF and palm oil carotene exhibited potent natural antioxidant properties in modulating inflammatory reactions in asthmatic. Hence, it can be considered as alternative approach to treat asthma over dexamethasone drug through their natural therapeutic effects.

Several limitations were identified in this study wherein the rats used might not resemble the actual pathogenesis of asthma in humans, even though these OVA-Challenged Asthmatic Brown Norway rats were used according to the physiology of their lungs with humans. Besides, the amount of TRF and carotene used in these rats may not be the same as required for asthmatic patients. Hence, further investigation is required to clarify the roles of TRF and carotene in inflammatory signaling pathways in the pathogenesis of asthma in human subjects. Collectively, the present findings provide clear evidence to suggest the potential benefits of the phytonutrients tocotrienol and carotene to be consumed as remedial agents in asthmatic disease.

## 4. Materials and Methods

### 4.1. Animals and Diets

Specific pathogen-free female Brown Norway rats (7 weeks of age) were purchased from Sterling Ascent Sdn Bhd, Penang, Malaysia. Animals were subjected to 12 h light/dark cycle air-conditioned rooms. The ethical approval (Ref number: UPM/IACUC/AUP-R061/2015) for animal use protocols was obtained from the Institutional Animal Care and Use Committee University Putra Malaysia (IACUC/101) on 23 November, 2015. Rats were housed at a maximum of two per cage. All rats were fed with altromin (Altromin 1324 FORTI), a maintenance diet for rats and mice that is fortified with 25,000 IU Vitamin A, 1000 IU Vitamin D_3_ (cholecalciferol), and 125 mg Vitamin E (α-tocopherol). TRF from palm oil (70% tocotrienol and 30% tocopherol) was obtained from Sime Darby Biogenic Sdn. Bhd, Malaysia. Palm carotene was obtained from Carotino Sdn Bhd, Malaysia. Dexamethasone was obtained from Sigma Aldrich, USA. Standard enzyme linked immunosorbent assay (ELISA) kits used for the determination of rat IL-β, IL-6, IL-4, IL-13, TNF-α (Novateinbio, Woburn, Massachusetts, USA), CRP (eBioscience, Affymetrix, Santa Clara, California, USA), IgE, LTB-4, and LTB-5 (MyBioSource, San Diego, California, USA) were utilized.

### 4.2. Sensitization and Allergen Challenge

Fifty female Brown Norway rats were randomly assigned into five experimental groups (10 rats per group based on power of calculation) according to the study design conducted by Wagner et al. [26]. Female rats were chosen because asthma attacks occur more frequently and are more critical in women than men [34]. The groups were as follows: normal control (group 1), positive control: dexamethasone treatment (30 mg/kg body weight) (group 2), negative control: reverse osmosis water treatment (2 mL) (group 3), TRF 30 mg/kg body weight (group 4), and carotene 30 mg/kg body weight (group 5). The doses of TRF, carotenes, and dexamethasone used in this study were chosen according to Raju et al. [35] and Ommen et al. [36], who showed beneficial progress in a rat model challenged with OVA. Throughout the present study, rats were placed in PVC locked cages and had access to food and water ad libitum. On day 0 and day 7, rats were sensitized with chicken albumin, which acts as an adjuvant by intraperitoneal injection. The chicken albumin was prepared by adding 2.5 mL sterile saline of a solution comprising 1 mg OVA mixed with 10 mg alum (aluminium potassium sulphate) [26]. After 14 days, rats were re-challenged with OVA for the four subsequent days (i.e., Days 14, 15, 16, and 17) by intranasal instillation (IN). The procedure was carried out by injecting 25 µL of OVA (0.4 mg/mL in saline) into individual nasal passages. Beginning at 8 h after the last challenge, treatment was initiated wherein rats were administered oral gavage, 30 mg/kg body weight of TRF, carotene, or dexamethasone. Body weight of the rats was monitored daily. The treatment was administered daily for four consecutive days via the oral gavage method until day 22. Finally, the animals were sacrificed and tissues were gathered on Day 22 (Figure 1).

### 4.3. Collection of Plasma, BAL, and Tissue Harvest

Rats were euthanized with pentobarbital, followed by blood withdrawal via cardiac puncture and collection in heparinized tubes. Whole blood was acquired by centrifugation at 2800× *g* at 4 °C for 10 min, and was stored at −80 °C. After blood collection, lungs were lavaged twice with 2 mL of normal saline (0.9%). BAL fluids were promptly placed on ice and centrifuged at 500× *g*. An aliquot (10 µL) of BAL cell suspension was cyto-centrifuged (3000 rpm) and recolored with Wright-Giemsa dye for determination of differential cells. Plasma obtained from centrifugation of whole blood was utilized for the estimation of cytokines.

### 4.4. Necropsy and Tissue Preparation

BAL fluids from the right lung lobes were withdrawn by adding 3 mL of sterile saline while the bronchus of the left lung was temporarily clamped. The collected BAL fluids from left and right lung lobes were fixed in 10% formalin. After 2 days, the BAL fluids were decalcified in 10% ethylenediaminetetraacetic acid (EDTA) solution for detoxification. Lung tissues were then dried, treated, and fixed with paraffin wax. Consecutive sections of 5 µm from each block were prepared, marked with hematoxylin and eosin, and observed using a light microscope.

### 4.5. Hematology

The blood from each animal was collected in EDTA tubes and immediately mixed with the anticoagulant. After collection, whole blood was used for the complete blood count. Diluted blood cells were applied on a hemocytometer for cell counting [37]. Packed cell volume (PCV) was tested by centrifugation at 3000 *g* for 30 min [38]. Hemoglobin (Hb) content was studied by cyanide-free hemoglobin determination [37]. Mean cell volume (MCV), mean corpuscular hemoglobin (MCH), and mean corpuscular hemoglobin concentration (MCHC) were calculated using the following formula [37]: MCV= (PCV/RBC) ×10; MCH= (Hb/ RBC) ×10; MCHC= (Hb/PVC) ×100.) This was all performed in a hematology analyzer (Model number DxH 900, Beckman Coulter, Miami, FL, USA).

### 4.6. Cytokine, CRP, IgE, LTD4, and LTD5 Concentration Level in Serum and BAL Fluid

ELISAs were applied to determine the concentrations of cytokines in the plasma and BAL fluids according to manufacturer instructions. IL-β, IL-6, IL-4, IL-13, TNF-α, CRP, IgE, LTB-4, and LTB-5 were analyzed using an ELISA plate reader (Synergy H1 Hybrid Multi-Mode Reader, Model no 270757, HIMF, Biotek, Winooski, VT, USA).

### 4.7. Statistical Analysis

All data obtained were analyzed using the Statistical Package for the Social Sciences (SPSS) version 20 (SPSS Inc., USA). All data are expressed as the means ± standard deviation (SD). One-way analysis of variance (ANOVA) followed by Bonferroni’s post-hoc test was used for comparing the experimental means between different experimental and control groups. A *P*-value of <0.05 was considered indicative of statistically significance.
